# Cellular immune response to intrastriatally implanted allogeneic bone marrow stromal cells in a rat model of Parkinson's disease

**DOI:** 10.1186/1742-2094-6-17

**Published:** 2009-06-05

**Authors:** Dianne M Camp, David A Loeffler, Diane M Farrah, Jade N Borneman, Peter A LeWitt

**Affiliations:** 1 Division of Neurology, William Beaumont Hospital Research Institute, Royal Oak, Michigan 48073, USA; 2 Cognate Bioservices, Baltimore, Maryland, USA; 3 Department of Neurology, Henry Ford Hospital, Detroit, Michigan 48034, USA

## Abstract

**Background:**

Marrow stromal cells (MSC), the non-hematopoietic precursor cells in bone marrow, are being investigated for therapeutic potential in CNS disorders. Although *in vitro *studies have suggested that MSC may be immunologically inert, their immunogenicity following transplantation into allogeneic recipients is unclear. The primary objective of this study was to investigate the cellular immune response to MSC injected into the striatum of allogeneic recipients (6-hydroxydopamine [6-OHDA]-hemilesioned rats, an animal model of Parkinson's disease [PD]), and the secondary objective was to determine the ability of these cells to prevent nigrostriatal dopamine depletion and associated motor deficits in these animals.

**Methods:**

5-Bromo-2-deoxyuridine (BrdU) – labeled MSC from two allogeneic sources (Wistar and ACI rats) were implanted into the striatum of adult Wistar rats at the same time as 6-OHDA was administered into the substantia nigra. Behavioral tests were administered one to two weeks before and 16–20 days after 6-OHDA lesioning and MSC transplantation. Immunocytochemical staining for T helper and T cytotoxic lymphocytes, microglia/macrophages, and major histocompatibility class I and II antigens was performed on post-transplantation days 22–24. MSC were detected with an anti-BrdU antibody.

**Results:**

Tissue injury due to the transplantation procedure produced a localized cellular immune response. Unexpectedly, both sources of allogeneic MSC generated robust cellular immune responses in the host striatum; the extent of this response was similar in the two allograft systems. Despite these immune responses, BrdU^+ ^cells (presumptive MSC) remained in the striatum of all animals that received MSC. The numbers of remaining MSC tended to be increased (*p *= 0.055) in rats receiving Wistar MSC versus those receiving ACI MSC. MSC administration did not prevent behavioral deficits or dopamine depletion in the 6-OHDA-lesioned animals.

**Conclusion:**

MSC, when implanted into the striatum of allogeneic animals, provoke a marked immune response which is not sufficient to clear these cells by 22–24 days post-transplantation. In the experimental paradigm in this study, MSC did not prevent nigrostriatal dopamine depletion and its associated behavioral deficits. Additional studies are indicated to clarify the effects of this immune response on MSC survival and function before initiating trials with these cells in patients with PD or other neurodegenerative disorders.

## Background

The standard treatment for Parkinson's disease (PD) for several decades has been the dopamine precursor levodopa. However, long-term administration of levodopa eventually results in decreased efficacy and the emergence of side effects including dyskinesias and psychoses. Interest has therefore grown in cellular restoration of striatal dopaminergic innervation in PD patients. Intrastriatal implantation of fetal mesencephalic tissue has resulted in long-term reductions of motor deficits [1,2] and normalization of striatal dopamine levels [3] in some patients, but this approach is limited by ethical and practical concerns, as well as the development of dyskinesias. In addition, although the brain was traditionally regarded as "immunologically privileged," long-term survival of fetal mesencephalic grafts in the adult brain is poor due to immune rejection and possibly other mechanisms [4]. More recent studies have suggested that stem cells may be useful in treating PD [5-7]. These cells offer significant advantages over fetal tissue for treatment of PD, including their ability to be expanded in culture and receive transfected genes and their potential for migration and differentiation in host tissue [8]. However, ethical and logistical issues similar to those for fetal mesencephalon transplantation also apply to human embryonic stem cell therapy.

Bone marrow stromal cells (MSC), the non-hematopoietic precursor cells (i.e. mesenchymal stem and progenitor cells) in bone marrow, offer an alternative source of cells for treatment of neurodegenerative diseases and central nervous system (CNS) injury. These cells normally differentiate into bone, cartilage, and adipose tissue [9], but can be experimentally induced to differentiate into cells with surface markers characteristic of neurons [10,11]. When injected into the brain or administered systemically, MSC can migrate to sites of injury, proliferate, and engraft [12-15]. These cells offer several advantages over other sources of stem-like precursor cells as therapy for PD: they are easily harvested, isolated, and purified, can be produced in large quantities, and their use does not pose ethical concerns. Potential roles for MSC in treatment of PD include their use as vectors for delivery of gene products to sites of tissue injury [16-19], facilitation of recovery from neuronal damage by replacing injured and/or lost cells [20-22], and production of trophic factors promoting survival and regeneration of host tissue [23-26]. In support of these therapeutic concepts, modest improvements in neurological function have been reported following MSC administration in animal models of PD, stroke, and acute CNS injury [26-29]. Although patients could be treated with their own MSC (an autologous transplant) to avoid possible immune rejection of the administered cells, the use of previously harvested and *in vitro*-expanded MSC from other individuals (an allogeneic transplant) would avoid the necessity for the patient to undergo bone marrow aspiration, and would allow rapid administration of previously expanded (and, if warranted, genetically modified) MSC.

Surprisingly little is known about the CNS immune response to locally administered MSC. However, a large number of *in vitro *studies have suggested that MSC possess immunosuppressive properties and, therefore, may not induce an immune response (for review see [30]). For example, MSC inhibit T cell proliferation induced by allogeneic lymphocytes or mitogens [31-33] and inhibit both naïve and memory T cell activation [34]. These effects are independent of major histocompatibility complex (MHC) expression (MSC express MHC class I antigens and low levels of MHC class II antigens and co-stimulatory molecules) [33], and have been attributed, in part, to the secretion of soluble factors that inhibit production of inflammatory cytokines and increase production of immunosuppressive ones [32,35]. However, whether MSC exert these immunosuppressive effects *in vivo *is unclear. In support of this possibility, MSC transplantation prolongs survival of skin allografts in baboons [31], allows growth of tumor cells otherwise rejected by immunocompetent recipients [36], and attenuates acute graft-versus-host disease after allogeneic bone marrow transplantation [37,38]. Conversely, other studies have reported that MSC do not prevent the graft-versus-host response [39,40]. There also is no consensus as to whether MSC are immunogenic *in vivo*. In some studies, transplantation of allogeneic or xenogeneic MSC into immunocompetent animals was not associated with a detectable host immune response [12,15,25,41,42], whereas in others, an immune response and/or rejection of MSC were observed [39,43-47]. Survival of allogeneic MSC transplanted into the CNS is low [12,28,45], suggesting that they may be targeted for removal by the host immune system.

The objectives of the present study were to characterize the local cellular immune response to allogeneic MSC after transplantation into the striatum of unilaterally 6-hydroxydopamine (6-OHDA) – lesioned rats, a well-characterized animal model of PD, as well as the ability of these cells to improve motor function in this model. Two types of allografts were employed: (1) MSC obtained from the inbred ACI strain were implanted into Wistar rats, and (2) Wistar MSC were implanted into Wistar rats from a different breeding colony. Because Wistar is an outbred, random-bred strain, tissues exchanged between Wistar rats are allografts [48]. Rejection of fetal CNS grafts occurs with both types of allografts, although it occurs more rapidly in grafts between unrelated inbred strains than in grafts between rats of the same outbred strain (i.e. Wistar-to-Wistar or Sprague-Dawley-to-Sprague-Dawley) [49]. We hypothesized that if a cellular immune response occurs in the brain following local administration of allogeneic MSC, then the extent of this response might differ between these two allogeneic systems.

## Methods

### Animal welfare

The protocols used in this study were approved by the Institutional Animal Care and Use Committee at William Beaumont Hospital, Royal Oak, Michigan, USA

### Animals and unilateral 6-OHDA lesioning

Adult female Wistar rats (Crl:WI, Charles River Laboratories, Raleigh, North Carolina, USA), weighing 200–225 g were housed in pairs with free access to food and water. After completion of baseline behavioral testing (see below), animals were anesthetized with sodium pentobarbital (25–40 mg/kg, i.p.) and received a unilateral stereotaxic injection of 6-OHDA hydrobromide (Sigma Chemical Company, St. Louis, Missouri, USA) into the substantia nigra. Briefly, 4 μg of 6-OHDA in 2 μl of saline-0.1% ascorbate solution was infused over a 4 min period via a 30 gauge cannula at the following coordinates [50]: 5.0 mm posterior to bregma, 2.0 mm lateral to the midline, and 7.4 mm ventral from the skull surface. Our intention was to generate partial, rather than complete, lesions, similar to the extent of nigrostriatal dopamine depletion in PD patients; our preliminary studies found that this concentration of 6-OHDA produced a 70–95% loss of striatal dopamine in most animals. The cannula was left in place for 2 min following infusion to allow diffusion of the solution. Desipramine (Sigma, 15 mg/kg, i.p.) was administered 30 to 60 min before 6-OHDA to protect noradrenergic terminals.

### MSC preparation for transplantation

Male rat MSC from the outbred Wistar strain (Hsd:WI, Harlan Sprague-Dawley, Inc.) and the inbred ACI strain (ACI, Harlan Sprague-Dawley, Inc.) were provided by Theradigm, Inc. (Baltimore, Maryland, USA) as frozen vials of cells (passage #4). Flow cytometric data provided by Theradigm, Inc. indicated that these MSC were positive for CD44 (ACI MSC 87.3%, Wistar MSC 76.9%), CD73 (ACI MSC 53.2%, Wistar MSC 63.8%), CD90 (ACI MSC 98.1%, Wistar MSC 96.6%), and MHC class I antigens (no data available for ACI MSC; Wistar MSC 79.5%), and negative for CD31, CD45, and MHC class II antigens. These results are similar to those published previously for rat and human MSC [51-53]. Cells were rapidly thawed, then cultured in alpha minimal essential medium (MEM; Gibco, Invitrogen Corporation, Carlsbad, California, USA) containing L-glutamine, supplemented with 10% fetal bovine serum (FBS, provided by Theradigm, Inc.) and antibiotic-antimycotic (Penicillin, Streptomycin, and Amphotericin B; Gibco). Cells were cultured at 37°C in 5% CO_2 _for 8–9 days, replacing the culture medium twice. 5-Bromo-2-deoxyuridine (BrdU; Calbiochem; 10 μM) was added to the medium 72 hr before the cells were harvested. BrdU labeling efficiency exceeded 95% for both strains of MSC (data not shown). For transplantation, MSC were detached by brief incubation with 0.25% trypsin, washed once with phosphate buffered saline (PBS), then resuspended in PBS to 5 × 10^4 ^live cells/μl. Cell viability, estimated by trypan blue dye exclusion, ranged from 83–95%. Viability was similar for ACI- and Wistar-derived MSC. Cells were kept on ice and used within 1 hr.

### Intrastriatal transplantation of MSC

Four striatal sites on the same side as the 6-OHDA lesion were selected for MSC transplantation at the following coordinates [50]: (1) 0.6 mm rostral to bregma, 2.5 mm lateral to the midline, 4.5 mm and 6.0 mm ventral from the skull surface; and (2) 0.5 mm rostral to bregma, 3.5 mm lateral to the midline, 4.5 and 6.0 mm ventral from the skull surface. Immediately after 6-OHDA infusion, animals (n = 6/group) received a 4 μl suspension of MSC (~200,000 cells) into each of the sites using a 22 or 26 gauge Hamilton microsyringe attached to a microinjector unit on the stereotaxic apparatus. Cells were injected at a rate of 0.5 μl/min, and the needle was left in place for 2 min before slowly retracting it. Control animals (n = 6/group) included similarly lesioned rats receiving infusions of PBS rather than MSC, and non-lesioned, naïve rats (for behavioral studies).

### Behavioral procedures

Behavioral tests were administered one to two weeks prior to the day on which the 6-OHDA lesioning and MSC administration were performed, in order to establish a baseline for normal performance, and were repeated at 16–20 days following the lesioning/transplantation procedure. The tests were drug-induced rotational behavior [54], stepping test [55], limb use asymmetry test ("cylinder test," [56]), bilateral tactile stimulation test ("adhesive removal test," [56]), and rotarod test [57].

#### Drug-induced rotational behavior

Animals were tested for both apomorphine- and amphetamine-induced rotational behavior. Challenges with apomorphine and amphetamine assess different areas in the dopamine system: apomorphine stimulates dopaminergic receptors whereas amphetamine stimulates the release of dopamine [54]. Apomorphine-induced rotational behavior was measured for 60 min following a subcutaneous injection of apomorphine (Sigma; 0.05 mg/kg, in 0.9% saline-0.1% ascorbate solution). Amphetamine-induced rotational behavior was measured for 120 min following intraperitoneal injection of d-amphetamine sulfate (Sigma; 1.5 mg/kg, in 0.9% saline).

#### Forelimb stepping test

The animal was held by the experimenter so that one forepaw rested on a smooth table surface while the other limbs were restrained. The number of adjusting steps was counted while the animal was moved sideways along the table surface (90 cm in 5 sec) in the forehand direction, with both forelimbs. A reduced stepping rate with the contralateral limb occurs with a 50–60% loss of dopaminergic neurons in the substantia nigra, and the level of performance gradually diminishes with larger lesions [58].

#### Cylinder test

Animals were placed in a 25 cm diameter (46 cm high) transparent cylinder and behavior was videotaped in the dark for 5 min. Rearing behavior was analyzed, and the number of contacts made by each forepaw with the cylinder wall was determined from the videotapes.

#### Bilateral tactile stimulation test

Animals were tested for unilateral sensorimotor impairments before and after MSC implantation using the adhesive-removal ("sticky label") sensorimotor test [56]. Two small pieces of adhesive-backed paper (1.2 cm in diameter) were used as tactile stimuli on the distal-radial region of each forelimb, and the time required for the animal to remove each stimulus from the forelimbs was recorded.

#### Rotarod test

The rotarod apparatus (Economex Accelerating Rota-Rod, Columbus Instruments, Columbus, Ohio, USA) measures the time that an animal is able to remain on a rod rotating at different speeds. Motor deficits related to limb dexterity and speed of movement lead to the animal falling off the rotating cylinder; thus, this test is useful for evaluation of motor impairments in animal models of Parkinsonism. Animals were placed on the rod rotating at an accelerating speed from 0 to 48 rpm in 480 sec. The time that the animal remained on the rod and the maximal speed reached prior to falling were recorded.

### Histology and immunohistochemistry

Animals were sacrificed 22 to 24 days after MSC transplantation, following behavioral testing on post-transplantation days 16–20. The gap of a few days between behavioral testing and sacrifice was necessitated by the staggering of the lesioning/transplantation procedures over several days, with subsequent staggering of behavioral testing to coincide with the surgery dates. Following induction of deep anesthesia by pentobarbital (60–80 mg/kg, i.p.), animals were intracardially perfused with 0.1 M PBS, followed by 4% paraformaldehyde in PBS. Brains were removed and post-fixed for 4 h in the same fixative, then cryoprotected in 0.1 M PBS with 20% sucrose overnight on ice. Each brain was cut into two coronal pieces, with one piece including the striatum and the other piece including the substantia nigra, then rapidly frozen on dry ice and stored at -70°C. Striatal and substantia nigra sections were subsequently cut at 20 μm thickness on a cryostat. Sections were collected, thaw-mounted on microscope slides (Superfrost Plus, Cardinal Health, McGaw Park, Illinois, USA) and stored at -20°C.

#### BrdU immunohistochemistry

Every ninth section throughout the striatum was stained for BrdU. After three washes in 0.1 M Tris buffered saline (TBS), pH 7.6, with 0.1% Triton X-100 (TBS-T), sections were pre-treated for 30 min in boiling citrate buffer, pH 6.0 (Antigen Unmasking Solution, Vector Laboratories, Burlingame, California, USA), then cooled for 20 min at room temperature in the same solution. Sections were washed in TBS-T, then treated with 3% hydrogen peroxide and 10% methanol in TBS for 30 min to quench endogenous peroxidase activity. After three washes in TBS-T, nonspecific binding of immunoglobulins was blocked by 1 hr incubation with 10% normal horse serum (NHS) in TBS-T containing 2% bovine serum albumin (BSA). Monoclonal anti-BrdU antibody (Calbiochem, San Diego, California, USA) was diluted 1:200 in TBS-T/1% NHS/2% BSA, then applied to the sections and allowed to incubate overnight at 4°C. Sections were then washed with TBS and incubated with biotinylated, rat-preabsorbed, horse anti-mouse IgG (Vector, 1:200) in TBS-T/1% NHS/2% BSA for 1 hr. Following washes in TBS, sections were incubated for 1 hr with avidin-biotin horseradish peroxidase complex (Vectastain Elite ABC kit, Vector) diluted 1:100 in 0.1 M TBS. After additional washes in TBS, the sections were developed using 3,3-diaminobenzidine with nickel enhancement (DAB kit, Vector). Slides were rinsed in distilled water, dehydrated through graded concentrations of ethanol, cleared in xylene and coverslipped using Cytoseal 60 mounting medium (Richard-Allan Scientific, Kalamazoo, MI). Negative controls consisted of substituting normal mouse IgG (Vector) for the primary antibody, using the same protein concentration as for the primary antibody. No immunoreactivity was present on these negative control slides. Staining of the contralateral striatum served as an additional negative control.

#### MHC I and II, CR-3, CD4, and CD8 immunohistochemistry

BrdU staining (see above) was used to determine optimal sections for evaluating the host cellular immune response to MSC. Each antibody for detection of immune cell surface antigens was applied to three sections per animal. The three sections were not equally spaced, but were chosen to include the regions where BrdU immunoreactivity was moderate to strong. The interval between sections averaged approximately 320 μm, with a range of 140 to 520 μm. The following monoclonal antibodies were used (all from Serotec, Raleigh, NC): OX18 (1:500), which recognizes rat MHC class I antigen, OX6 (1:500), which recognizes rat MHC class II antigen appearing on B lymphocytes, certain epithelial cells, dendritic cells, some macrophages, and activated microglia (commonly used for microglial detection), OX42 (1:200), which recognizes the CR3 receptor expressed on most macrophages, resting and activated microglia, and monocytes (commonly used for microglial detection), W3/25 (1:500), which recognizes the CD4 antigen appearing on T-helper lymphocytes, microglia and some macrophages, and OX8 (1:400), which recognizes the CD8 antigen appearing primarily on cytotoxic T-lymphocytes. These antibodies have been used in previous investigations of the immune response following transplantation of other types of cells into the brain [59-61]. Procedures were as described for BrdU immunocytochemistry, except that sections were not pre-treated with citrate buffer, TBS molarity was 0.05 M, and antibody dilution buffer did not include BSA.

#### Tyrosine hydroxylase (TH) immunohistochemistry

Striatal and substantia nigra sections were stained for TH using similar procedures to those described for MHC antigens, except that 0.3% hydrogen peroxide/2% NHS in TBS was used to quench endogenous peroxidase activity. The anti-TH monoclonal antibody (LNC-1, provided by Dr. G. Kapatos, Wayne State University, Detroit, MI) was diluted to 1:3,000.

### Quantitation of immunocytochemical staining

Immunocytochemical staining was assessed using a Nikon Eclipse E400 microscope with a 10× or 40× objective and an Optronics DEI-750 video camera connected to a computerized image analysis system (BIOQUANT-R&M Biometrics, Inc, Nashville, Tennessee, USA). Immunoreactive staining for MHC I and II, CR-3, and CD4 was quantified in terms of both its intensity (i.e. relative optical density) and the percent area occupied in the region of interest. To quantify immunostaining intensity, optical density was measured in four equivalent fields (75 μm^2 ^per field) adjacent to the injection tract where immunoreactivity was most intense, and the measurements obtained for the four fields were averaged for each section. Densitometric measurements were corrected for background intensity. Optical density did not differ between treatment groups for any of the cell surface antigens, therefore these data are not presented. To measure the percent area occupied by immunoreactivity, a threshold for positive staining was determined for each cell surface antigen, then this setting was used on all sections to measure the area of the thresholded pixels in the delineated region of interest. The "invert threshold" function was used to determine the area of the non-thresholded pixels in the same region. This was necessary in order to determine the percentage of the region of interest occupied by immunoreactive pixels. The highest measurement for percent immunoreactivity among the three slides was used for statistical analysis. The striatum was delineated by the following boundaries: medial: lateral ventricle; dorsal and lateral: corpus callosum, and ventral: anterior commissure. Because individual cells stained for BrdU or CD8 could be visualized, the total number of these cells was counted on 40× images. As a relative estimate of the number of remaining MSC in the striatum, the number of BrdU-positive cells was counted in every ninth section throughout the striatum and then the counts from these sections were totaled for each striatum. To quantitate TH immunoreactivity in the striatum, the intact (contralateral) striatum was used to determine the threshold for what was considered to be positive TH staining, and then the area of the pixels that exceeded this threshold was measured for both striata. The same method was used to determine TH immunoreactivity in the substantia nigra: the threshold for positive immunoreactivity was determined for the substantia nigra on the non-lesioned side, then the area of the thresholded pixels was measured for both sides of the nigra. TH immunoreactivity in the substantia nigra was evaluated in the pars compacta and pars reticulata, but not the ventral tegmental area. All measurements were made in a blinded fashion.

### Statistics

Nonparametric statistics including the Kruskall-Wallis test and the Wilcoxon Two-Sample test were performed on the immunocytochemical staining data and on the behavioral data using SAS statistical software (SAS, Cary, North Carolina, USA) to evaluate treatment effects. The level of significance was set at *p *< 0.05 for all analyses. Spearman's rank correlation coefficients were calculated to assess the strength of association between the different host immune markers, as well as the strength of association between the number of striatal BrdU^+ ^cells and the host cellular immune markers.

## Results

### Behavior

**Figure 1:**
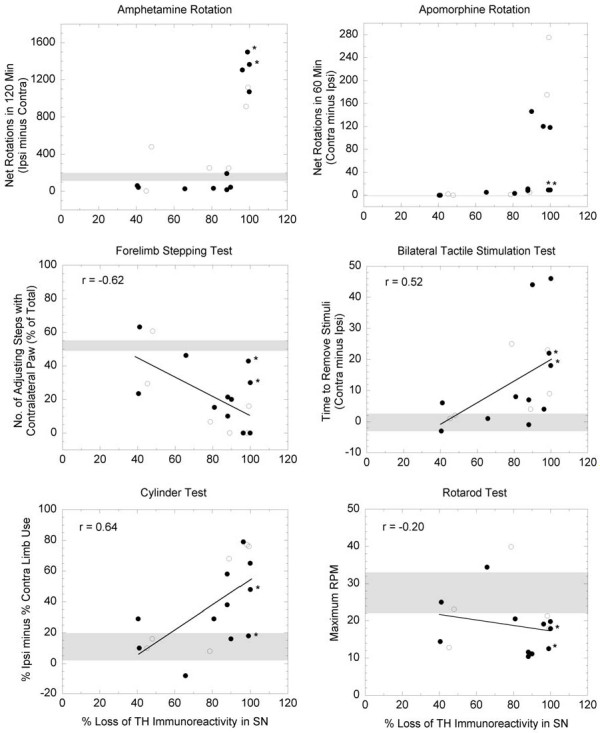
**Scatter plots of individual behavioral scores**. Relationship between lesion severity (percent loss of tyrosine hydroxylase [TH] immunoreactivity in the substantia nigra [SN]) and behavioral scores in PBS-treated (open circles, n = 6) and MSC-transplanted (closed circles, n = 11) animals. The behavioral performance of nonlesioned naive rats is indicated by the shaded region (mean ± SEM). A linear regression analysis was performed on the scatter plots (solid line); correlation coefficients are listed for each analysis. Amphetamine- and apomorphine-induced rotation was not linearly related to lesion severity, therefore no linear regression is shown for these plots. Two of the MSC-transplanted animals (indicated by asterisks) with nearly complete nigral lesions performed more similarly to animals with partial lesions in some of the behavioral tests.

There was considerable variability in behavioral scores within treatment groups, most likely because of the varying loss of nigrostriatal dopamine neurons within each group. Evaluation for statistical differences between treatment groups using the Kruskal-Wallis test was not useful because of the large variation in behavioral deficits and small sample sizes. (The sample sizes were chosen on the basis of a power analysis from data from a pilot investigation performed in our laboratory; however, the lesion size varied more in the present study than it did in the pilot study.) Data are therefore presented in Fig. 1 as scatter plots of the individual behavioral scores as a function of the degree of lesioning (i.e. percent loss of TH immunoreactivity in the substantia nigra). An analysis of covariance model was fit to evaluate the relationship between lesion severity and performance on each behavioral test. The slope of the line relating the behavioral scores to lesion severity did not statistically differ between MSC- and PBS-treated animals; however, lesion severity was a significant predictor of forelimb stepping and cylinder test scores. Vigorous amphetamine- and apomorphine-induced turning was seen only in those animals with nearly complete dopaminergic lesions (> 95% loss of substantia nigra TH immunoreactivity). However, two of the three rats that had nearly complete dopaminergic lesions and that received Wistar-derived MSC did not rotate following apomorphine challenge. These animals also had smaller deficits on the stepping and cylinders tests than the other animals with similar-sized lesions. Interestingly, these two animals had the highest numbers of striatal BrdU-labeled MSC among all of the transplanted animals (15,119 and 13,005 BrdU^+ ^cells vs. 3,895 ± 1,099 [mean ± S.E.M.] for all other animals receiving MSC). The other rat in this group that rotated following apomorphine treatment had only 475 BrdU^+ ^cells. Except for this observation, MSC transplantation did not appear to reduce the deficits on any of the other behavioral tests.

### BrdU staining

**Figure 2:**
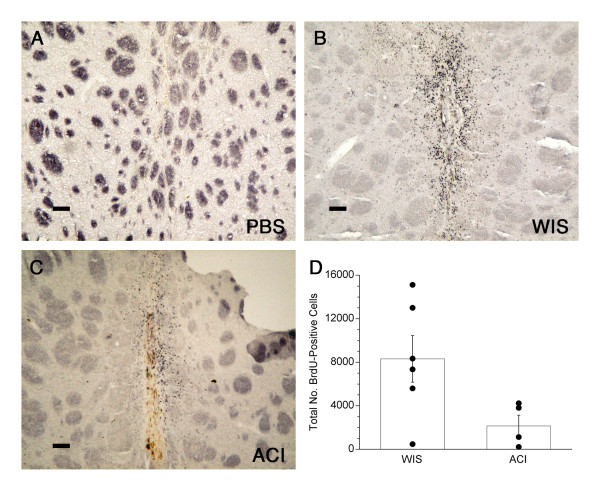
**BrdU immunoreactivity in striatum**. **(A) **Lack of immunoreactivity in the PBS-infused striatum. **(B-C) **BrdU^+ ^cells in Wistar rats receiving either Wistar (WIS) MSC or ACI MSC, respectively. **(D) **The numbers of BrdU^+ ^cells detected in every ninth 20 μm section throughout the striatum were totaled for each rat receiving either WIS MSC (n = 6) or ACI MSC (n = 4). Graph shows mean values ± SEM. Closed circles indicate values from individual animals. Scale bars = 80 μm.

BrdU-labeled MSC were easily discriminated from BrdU-labeled microglia and/or macrophages that had phagocytosed BrdU from degenerating MSC, as reported previously [43]; intense BrdU immunoreactivity was present in the nucleus of MSC, whereas lighter, cytoplasmic staining was observed in host cells [29]. Only cells with strong nuclear BrdU labeling were counted as MSC, as verified on images captured with the 40× objective. MSC were clearly detectable at 3 weeks post-transplantation (Fig. 2). The majority of MSC were clustered along the injection tract within the denervated striatum, although in a few cases moderate numbers of these cells were also found in the overlying cortex (around the injection tract) and in the corpus callosum. No BrdU immunoreactivity was detected in either the contralateral (nontransplanted) striatum or in histological sections from PBS-treated controls (Fig. 2A). In some animals, MSC were detected as far as 750 μm from the center of the injection tract (Fig. 2B). MSC counts (mean number of BrdU^+ ^cells ± S.E.M.) for each group are shown in Fig 2D. BrdU-positive cells were detected in the striatum in all animals receiving MSC, and the numbers of these cells detected in the 6-OHDA-lesioned Wistar rats receiving Wistar MSC tended to be greater than in those receiving ACI MSC (*p *= 0.055). Wistar MSC tended to migrate farther from the implantation site than did ACI MSC (Fig. 2B vs. 2C). The anterior-to-posterior extent of BrdU immunoreactivity, as estimated from staining every ninth section throughout the entire striatum, was 883 ± 101 μm (mean ± SEM) for Wistar MSC and 620 ± 83 μm for ACI MSC. The region of BrdU staining, as determined in the anterior-to-posterior direction, ranged from 220 μm to 1540 μm. BrdU staining was also performed on substantia nigra sections to determine whether intrastriatally delivered MSC migrated to the site of the 6-OHDA lesion. No BrdU^+ ^cells were detected in substantia nigra sections from MSC-treated animals.

### TH immunoreactivity

**Figure 3:**
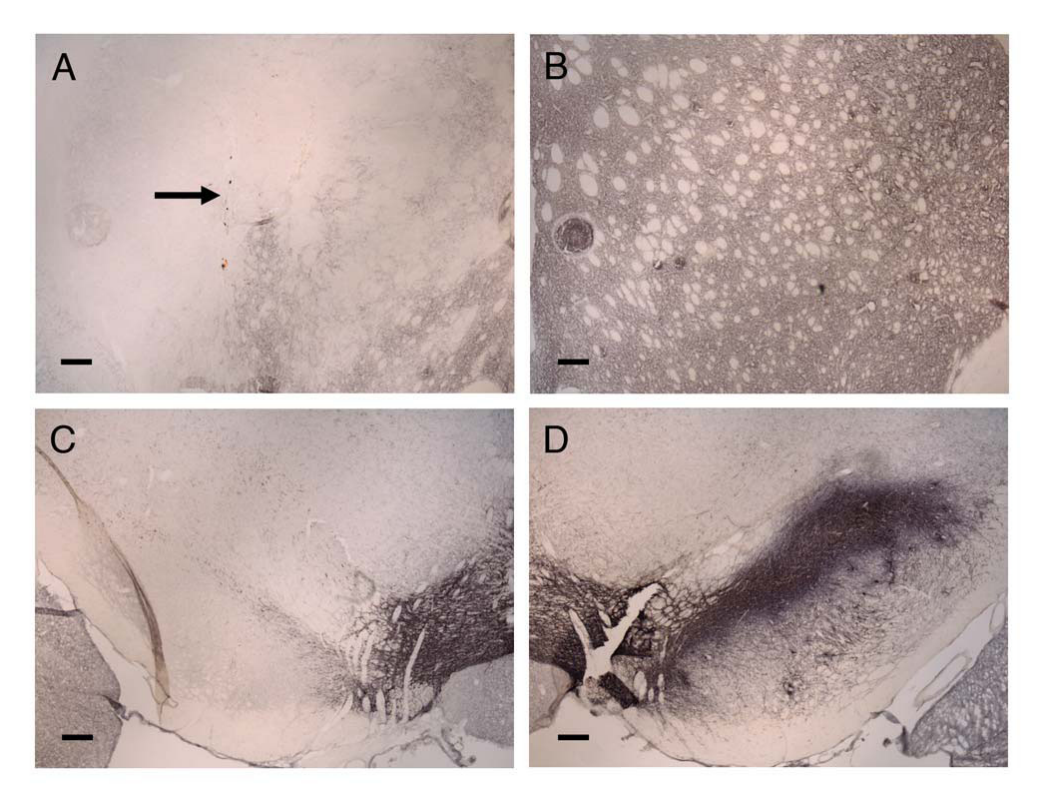
**TH immunoreactivity in striatum and substantia nigra**. Low power photomicrographs of TH immunostaining in the striatum **(A-B) **and the substantia nigra **(C-D) **from a rat receiving Wistar MSC. Similar staining was seen in animals receiving ACI MSC and in PBS-treated controls. Note the loss of TH immunoreactivity in the lesioned side (A, C) compared to the unlesioned side (B, D). Arrow indicates the needle track from the injection. Scale bars = 200 μm.

TH immunoreactivity was generally absent in the areas occupied by BrdU^+ ^cells, suggesting that the implanted MSC did not acquire the ability to synthesize TH, the rate-limiting enzyme for dopamine production (Fig. 3A). TH immunoreactivity in the lesioned striatum was variably reduced (by 4 to 99%) in comparison with the intact (contralateral) striatum (Fig 3B). TH immunoreactivity in the substantia nigra was highly correlated with TH immunoreactivity in the striatum (Spearman rho = 0.91, *p *< 0.0001; Fig. 3). There were no significant differences in the intensity of TH staining among the treatment groups.

### Cellular immune response in host striatum

#### Complement receptor 3 (CR3) immunoreactivity

**Figure 4:**
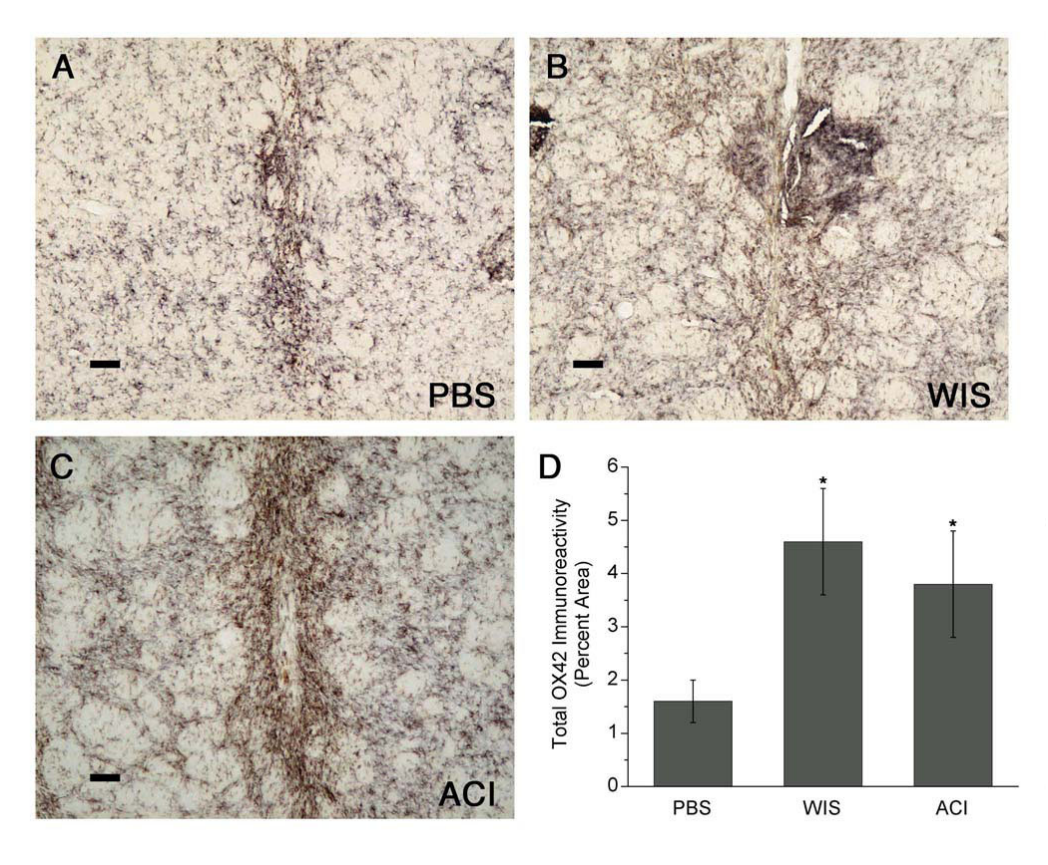
**CR3 (OX42) immunoreactivity in striatum**. **(A-C) **Widespread immunoreactivity to complement receptor 3 (CR3) in PBS-infused striatum, Wistar (WIS) MSC-implanted striatum, and ACI MSC-implanted striatum, respectively, in the same rats shown in Fig. 2. **(D) **The percent area (means ± SEM) of striatum (lesioned side) occupied by CR3 immunoreactivity in the MSC-injected groups (n = 4 – 6) was twice that of PBS-injected controls (n = 5; *p < 0.05). Scale bars = 80 μm.

Widespread immunoreactivity to OX42 was observed in all three groups (Fig. 4A–C). This was expected, because OX42 is expressed on resting microglia as well as reactive microglial cells and macrophages. Immunoreactivity in a remote area of the denervated striatum was used to determine the relative staining density of resting microglia, and this value was then subtracted from measurements made near the implantation site. Immunostaining was intense along the needle track, and large numbers of hypertrophic cells (most likely activated macrophages) were observed surrounding and within the implantation site. CR3 immunoreactivity (% immunoreactivity) in the MSC-injected groups was twice that of PBS-injected controls (*p *< 0.05) (Fig. 4D). There was no difference in CR3 immunoreactivity between the Wistar MSC- and ACI MSC-transplanted groups.

#### MHC immunoreactivity

**Figure 5:**
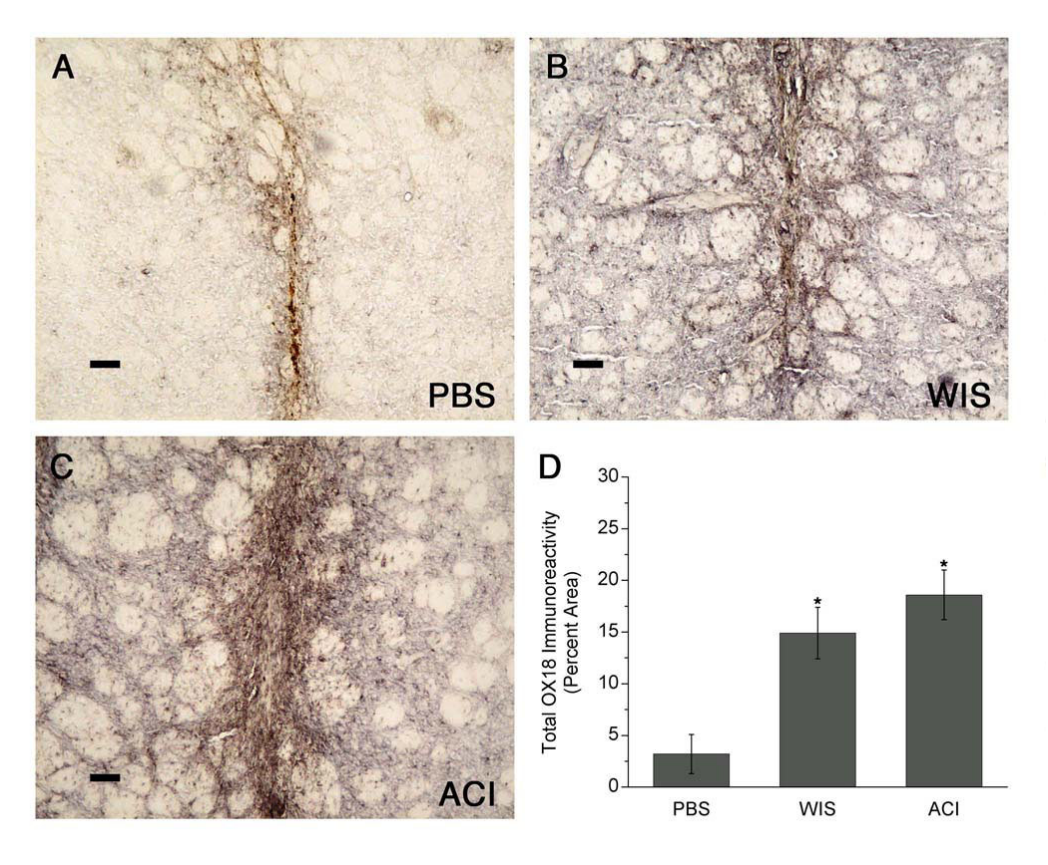
**MHC I (OX18) immunoreactivity in striatum**. **(A) **MHC class I staining in PBS controls was closely confined to the injection tract. **(B-C) **In animals receiving Wistar (WIS) MSC or ACI MSC (same rats shown in Fig. 2), a large area of the host striatum was infiltrated with MHC class I immunoreactive cells, although MHC expression was most intense surrounding and within the injection tract. **(D) **MHC class I staining occupied 15–20% of the striatum in MSC-transplanted animals, compared to less than 5% in PBS controls (graph shows means ± SEM; *p < 0.05). Scale bars = 80 μm.

**Figure 6:**
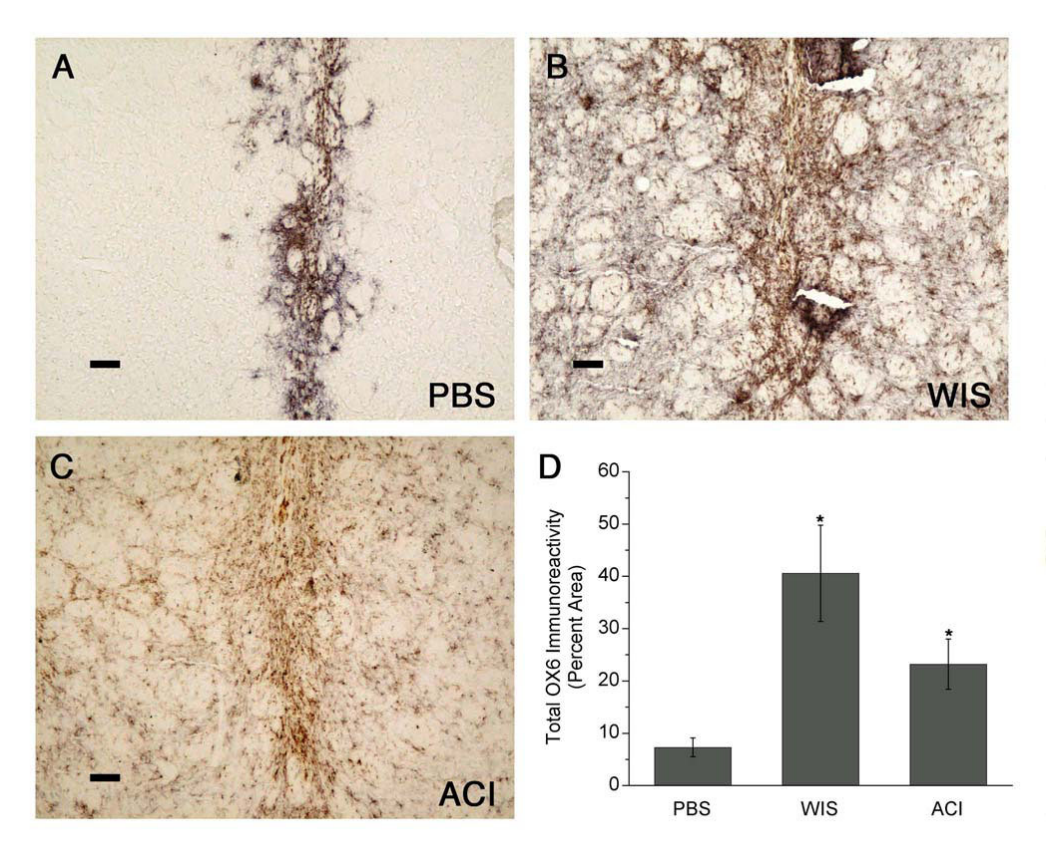
**MHC II (OX6) immunoreactivity in striatum**. **(A) **MHC class II staining in PBS controls was closely confined to the injection tract. **(B-C) **In animals receiving Wistar (WIS) MSC or ACI MSC (same rats shown in Fig. 2) MHC class II immunoreactivity was widespread in the striatum, but it was most intense surrounding and within the injection tract. **(D) **MHC class II staining was present in 20–40% of the striatum in MSC-transplanted animals, compared to less than 10% in PBS controls (graph shows means ± SEM; *p < 0.05). Scale bars = 80 μm.

In MSC-transplanted animals, a large area of the host striatum was infiltrated with MHC class I and class II immunoreactive cells, although MHC expression was most intense surrounding and within the injection tract (Figs. 5 and 6). In contrast, in the PBS control group, staining for these antigens was less intense and closely confined to the injection tract. Many MHC-immunoreactive cells around the injection tract exhibited microglia-like morphology. MHC class I immunoreactivity occupied 15–20% of the striatum in MSC-transplanted animals, compared to less than 5% in PBS controls (*p *< 0.05; Fig. 5D). MHC class II immunoreactivity was present in 20–40% of the striatum in MSC-transplanted animals (also *p *< 0.05 vs. PBS-injected controls; Fig. 6D). MHC immunoreactivity was not significantly different between the two MSC-transplanted groups.

#### Lymphocyte immunoreactivity

**Figure 7:**
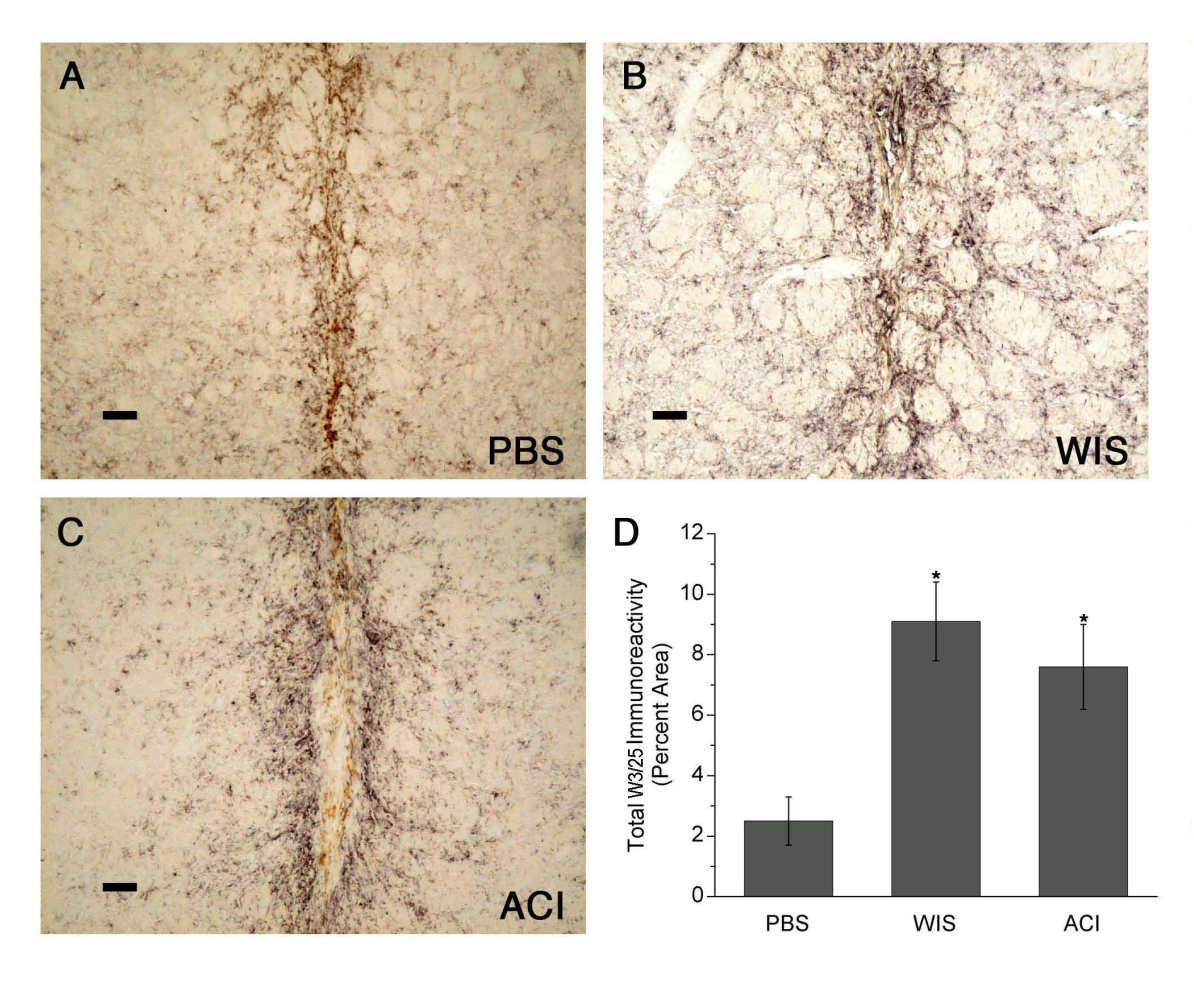
**CD4 (W3/25) immunoreactivity in striatum**. **(A) **The majority of CD4^+ ^cells in PBS-infused animals resembled microglia, although a few cells with lymphocytic morphology were also present. **(B-C)** CD4^+ ^cells with lymphocytic morphology were present in and around the needle tract in Wistar (WIS) MSC-treated and ACI MSC-treated animals (same rats shown in Fig. 2). **(D) **CD4 (W3/25) immunoreactivity was four-fold higher in MSC-transplanted animals than in PBS-infused controls *(*graph shows means ± SEM; *p < 0.05). Scale bars: = 80 μm.

The W3/25 antibody, which detects CD4^+ ^(T helper-inducer) lymphocytes, stained round cells with typical lymphocytic morphology as well as stellate cells resembling microglia, although the staining of these latter cells was weaker (Fig. 7A–C). A small population of W3/25^+ ^cells was present in and around the needle tract in both MSC-treated groups. In control animals, the majority of W3/25-immunoreactive cells surrounding the needle track resembled microglia, although a few cells with lymphocytic morphology were also present. W3/25 immunoreactivity was four-fold higher in MSC-transplanted animals than in PBS-infused controls *(p *< 0.05; Fig. 7D). There was no significant difference in W3/25 immunoreactivity between the two MSC-transplanted groups.

**Figure 8:**
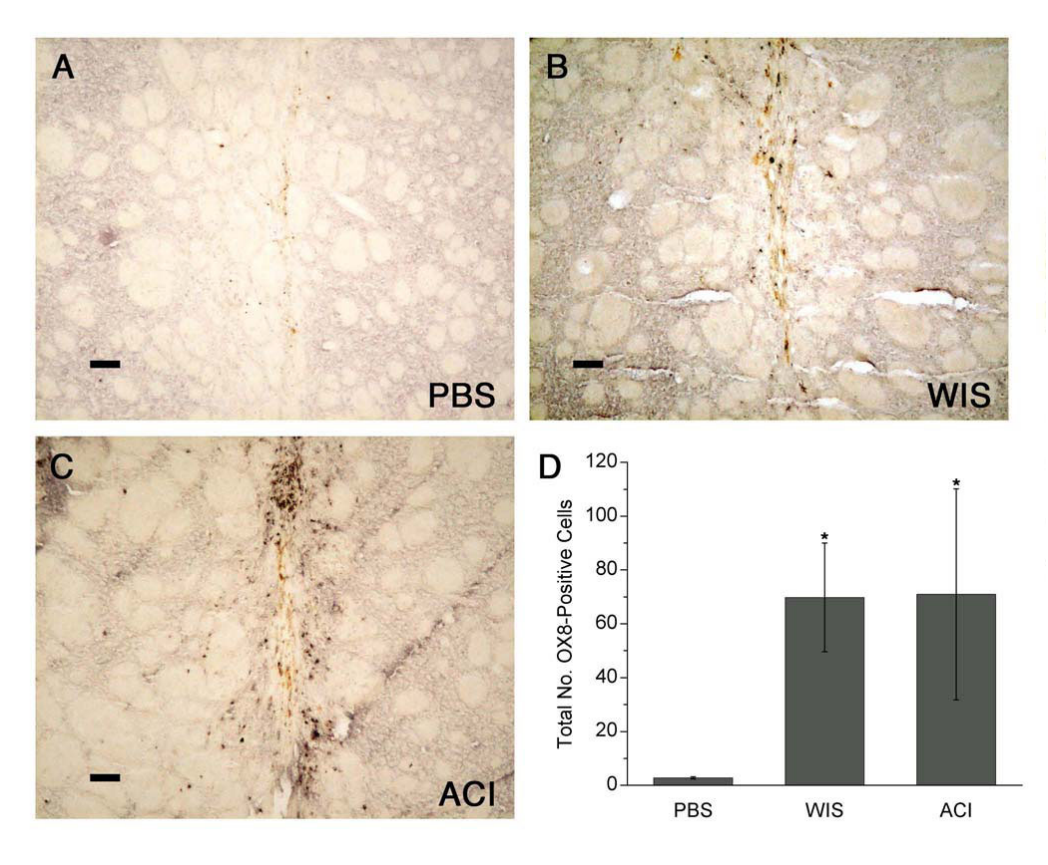
**CD8 (OX8) immunoreactivity in striatum**. **(A) **Little or no CD8 staining was observed in PBS control animals. **(B-C) **Few CD8^+ ^cells were detected in and around the injection tracts of Wistar MSC-treated and ACI MSC-treated animals (same rats shown in Fig. 2). **(D) **The numbers of CD8^+ ^(OX8) cells were significantly increased in the MSC-transplanted groups compared with PBS-injected controls *(*graph shows means ± SEM;*p < 0.05). Scale bars = 80 μm.

In contrast to W3/25, the OX8 antibody, which detects CD8^+ ^(T cytotoxic-suppressor) lymphocytes, stained only cells with typical lymphocytic morphology (Fig. 8A–C). There were far fewer OX8^+ ^than W3/25^+ ^lymphocytes in and around the injection tract. Few or no OX8^+ ^cells were detected in PBS control animals. OX8 staining was significantly increased in the MSC-transplanted groups compared with PBS-injected controls (Fig. 8D) (*p *< 0.05), and similar numbers of OX8^+ ^cells were present in the two groups of MSC-transplanted animals.

#### Correlational analyses

When data from the two groups of allogeneic recipients were combined, a moderate positive association was present between MHC class I and II staining in the striatum (r = 0.56). In addition, W3/25 (CD4) and OX8 (CD8) immunoreactivities were both moderately correlated with OX42 staining (r values = 0.60 and 0.52, respectively). No other correlations were present between the host cellular immune markers. A strong positive association was present between the numbers of striatal BrdU^+ ^cells and the extent of striatal MHC class II immunoreactivity (r = 0.77), but not for any other immune marker. No correlations were detected between the numbers of BrdU^+ ^cells and TH immunoreactivity in either the striatum or the substantia nigra.

## Discussion

In contrast to the *in vitro *studies cited earlier, our results indicate that a marked cellular immune response occurs when MSC are implanted into brains of allogeneic recipients. MHC class I- and class II-expressing cells and CD4^+ ^and CD8^+ ^lymphocytes were increased in the host striatum in both the ACI-to-Wistar and Wistar-to-Wistar systems. This study did not include Wistar-to-ACI MSC transplantation because the MHC differences would have been the same as for the ACI-to-Wistar transplant. Because rat strains differ in their ability to generate immune responses [62], we cannot rule out the possibility that strain-dependent differences in immune function may have affected the ACI and Wistar responses to allogeneic MSC. Outbred rats have frequently been used as recipients for CNS grafts (see, for example, references 41 and 49). Because ACI is an inbred strain, ACI-to-ACI transplantation would be a syngeneic system; this would provide no information about the local immune response to MSC transplanted into unrelated individuals (i.e. a "universal donor" paradigm), which was what we wished to model. Previous studies have reported that MSC transplanted into syngeneic recipients survive well and elicit only a short-lived host immune response after their transplantation into the CNS [47,63]. Although Wistar-to-Wistar transplants have been referred to as syngeneic [41], this is incorrect; because Wistar is an outbred strain, these transplants are actually allografts.

As stated earlier, cultured MSC typically express low to moderate levels of MHC class I antigens but generally do not express MHC class II or co-stimulatory molecules, and they secrete immunosuppressive cytokines [30]. An explanation for our contrasting finding of a strong cellular immune response in the brain following MSC administration is that MSC expression of MHC antigens and cytokine production may be altered *in vivo*. For example, it was recently reported that MSC lose their ability to downregulate T-cell immune responsiveness after allotransplantation [64]. The immunosuppressive properties of MSC may, in fact, be dose-dependent [33,34,65]. Another possible explanation for the discrepancy between the *in vitro *literature and our results could be tissue damage resulting from the transplantation procedure, which leads to an influx of inflammatory cells [59,61]. Brain injury disrupts the blood-brain barrier, permitting entrance of immune cells from peripheral blood [49,60]. The presence of these cells in the transplantation site could prime the brain for an immunological response to allogeneic MSC. In support of this possibility, transplantation procedures that minimize brain trauma and/or inflammation have been found to decrease graft rejection [66,67]. MSC administered intravenously have been suggested to avoid the host immune response [41], although the latter study examined only the systemic immune response (i.e. peripheral blood T cell priming, and antibody production) to these cells. Finally, mechanical injury to MSC at the time of implantation could also cause these cells to be seen as "foreign" by the host immune system. Although, as stated above, MSC viability was 83–95% prior to their transplantation, their viability was not assessed a second time after they were infused; therefore the possibility that some cells may have been damaged during this process cannot be ruled out.

MSC were still detected in the striatum at 3 weeks post-transplantation despite the presence of an active immune response to them. Previous studies have found that MSC allografts and xenografts can persist long-term in immunocompetent hosts [68,69], although immunosuppression may improve cell survival in these models [45,47]. Our results suggest that an active host immune response may not be successful in clearing these cells from the brain, as occurs following transplantation of other cell types [59,61,70]. Evaluation of additional time points would be necessary in order to clarify the relationship between the immune response in the CNS and long-term survival and function of allogeneic MSC.

No significant differences with regard to MSC survivability or host immune response were observed between the two types of allografts, although higher numbers of BrdU^+ ^cells tended to be present in the Wistar-to-Wistar transplants (*p *= 0.055). When data from the two groups of recipients were combined, the number of BrdU^+ ^cells was positively correlated with MHC class II immunoreactivity, suggesting that allogeneic MSC may upregulate MHC class II expression (presumably, on microglia) in the brain. The density of OX42^+ ^cells in MSC-treated animals was twice that of PBS-treated controls, indicating that the presence of MSC resulted in a local increase in microglia; this could have been due to secretion of chemotactic and/or proliferation-promoting factors by the MSC [71,72]. Our principal conclusion from this study is that, although allogeneic MSC stimulate a marked host cellular immune response in the brain, this response is not sufficient to clear the MSC, at least not by three weeks post-implantation. Studies investigating the host immune response at additional time points are required to determine the duration of this response and whether it eventually clears allogeneic MSC from the striatum. MSC survival might not, in fact, be correlated with the extent of the host immune response to them; if so, then this could explain why the two types of allografts showed similar levels of immune responses. Finally, the possibility cannot be ruled out that the trend for increased MSC survival in the Wistar-to-Wistar system could be due, in part, to differences in the numbers of viable MSC implanted. Although prepared in identical fashion, the suspensions of ACI MSC were more viscous than Wistar MSC suspensions, so more of these cells may have been injured during their infusion into the brain.

Microenvironmental conditions in the brain following acute injury, including the presence of oxygen radicals, excitotoxins, low concentrations of energy substrates, and/or alteration of local pH, may influence the survival and function of transplanted MSC. Further, the influx of inflammatory cells induced by tissue injury secondary to the surgical procedure may exacerbate the immune response to allogeneic cells. Introducing MSC into the brain by less invasive approaches, such as intravenous or intracarotid injections [13,41,69], or using techniques to minimize the degree of tissue injury might therefore improve their survival and function.

We cannot rule out that some of the BrdU^+ ^cells could be microglia which had phagocytosed MSC, as suggested by earlier studies [43,73], and this could have increased our MSC counts. However, several factors suggest that this is not case. We followed the procedure described by Li et al [29] to distinguish BrdU-labeled MSC from BrdU^+ ^phagocytic cells by their relative staining patterns: BrdU^+ ^MSC should display nuclear staining, whereas BrdU^+ ^phagocytes should have cytoplasmic staining. Both of these staining patterns were observed, therefore only cells with strong nuclear staining were counted as MSC. In addition, the distribution and pattern of staining for BrdU-labeled cells and microglia was not the same (e.g., compare the staining in Fig. 2B with Figs. 4B and 5B, which represent serial sections from the same rat). Double-labeling was not performed, therefore we do not know whether the BrdU^+ ^cells expressed the CR3 receptor, MHC I antigens, or MHC II antigens in vivo. However, our finding that the allogeneic MSC induced a strong host immune response in the striatum suggests that these cells did express MHC antigens, therefore our staining for MHC antigens could indicate MSC as well as microglia; further, some reports suggest that MSC may be capable of differentiating into glial cells, including microglia [15,27]. Because of these possibilities, even if cells staining for both BrdU and microglial antigens would be detected, this would not necessarily imply that BrdU transfer from MSC to microglia had occurred. The observation that two of the animals which received MSC had very low numbers of BrdU^+ ^cells in the striatum implies that transfer of BrdU to host cells was likely to have been negligible. In our preliminary studies we identified male donor MSC in female recipients by their Y-chromosome using *in situ *hybridization (data not shown). With this technique, MSC were detected in the implanted striatum of allogeneic 6-OHDA-hemilesioned rats after 13 weeks, supporting our findings in the present study that at least some MSC can remain in the brain despite a marked immune response to them. We chose to use BrdU immunostaining, rather than *in situ *hybridization, for detecting MSC in this study because of the more quantitative nature of BrdU staining.

Although the main goal of this study was to evaluate the immune response to intrastriatally-administered MSC, a secondary goal was to investigate the ability of these cells to prevent loss of nigrostriatal dopamine and associated behavioral deficits in an animal model of PD. The dopamine loss in our animals varied from 40% to 100%. Because of this variability, our study lacked sufficient statistical power to determine the therapeutic effects of MSC in these animals. Interestingly, the two animals with the highest numbers of striatal BrdU^+ ^MSC failed to rotate following apomorphine, although both animals had nearly complete 6-OHDA lesions. Apomorphine-induced rotation is used to identify animals with complete (or nearly complete) unilateral dopaminergic lesions; typically, only animals with greater than 90% loss of the nigrostriatal system rotate after administration of a low dose of apomorphine [74]. Although the data from these two animals suggest that MSC may be able to reduce behavioral deficits in animals with extensive dopamine depletion, studies with larger sample sizes will be required to determine if these observations can be confirmed. The low numbers of MSC in many of the animals may have contributed to the negative behavioral outcome in our study. Other studies evaluating the value of MSC in restoring function in the 6-OHDA rat model have reported varying results [16,18-22,75] with some of these studies reporting improvement only with genetically modified or neural-induced MSC [16,18,22]. A possible explanation for these differing results may be the numbers of MSC surviving in the brain after transplantation.

In contrast to previous studies, MSC were administered immediately following 6-OHDA lesioning. When 6-OHDA is administered into the medial forebrain bundle or substantia nigra, degeneration of dopaminergic neurons occurs within 24–72 hr [76]. Because the beneficial effects of MSC in the brain have been suggested to be due to their secretion of neurotrophic factors [23-25], we hypothesized that MSC administered at the time of lesioning might protect nigral neurons and their striatal terminals from the damaging effects of 6-OHDA. Such a strategy has the potential for therapeutic use in early PD to support and protect dopaminergic neurons against whatever factors are responsible for the ongoing neuronal loss. Transplants that are performed weeks after 6-OHDA lesioning, when dopaminergic cell loss has already been established, would more accurately model MSC administration to subjects with advanced PD; however, when early detection of the disease becomes possible, then effective neuroprotective treatments which can be administered prior to the extensive loss of dopamine neurons will be an important strategy. The same day lesion/transplant paradigm also allowed us to investigate whether intrastriatally transplanted MSC would migrate to the site of ongoing dopaminergic cell loss, i.e. the substantia nigra. Previous studies have indicated that MSC migrate to sites of tissue injury [13,14,77], perhaps due to locally-produced chemokines [78]. We hypothesized that in the acute 6-OHDA lesion model, chemoattraction of MSC would be greatest when dopamine neurons were dying. However, we found the loss of TH immunoreactivity in both striatum and substantia nigra to be similar between MSC-transplanted and PBS-control animals, and no BrdU^+ ^cells were detected in the substantia nigra. In contrast to our results with MSC, other types of cellular grafts have been shown to preserve nigrostriatal dopamine neurons and to reduce motor deficits when introduced at the time of 6-OHDA lesioning [7]. Additional studies are therefore warranted to determine the effects of MSC administered at different times in relation to 6-OHDA lesioning.

## Conclusion

MSC, when implanted into the brains of allogeneic rats, are detectable at post-implantation days 22–24 despite the presence of an active cellular immune response to them. Our findings are consistent with other studies indicating that allogeneic and xenogeneic MSC can provoke an immune response *in vivo *[39,43,44,46,47]. Whether this response contributed to the failure of MSC to reduce the behavioral deficits in the rats in this study that were extensively lesioned is not known. Further studies are indicated in which allogeneic MSC survival and therapeutic efficacy in animal models of PD will be compared between various routes of administration, at different time points, and in both immunocompetent and immunosuppressed animals.

## Competing interests

No competing interests exist for DMC, DAL, DMF, and PAL. JNB is employed by Cognate Bioservices, a company whose focus is on the commercialization of cell-based products.

## Authors' contributions

DMC designed the experiments, performed all animal-related procedures, analyzed the data, generated the figures, and wrote the manuscript. DAL assisted in growing and harvesting the MSC, assessment of staining, and writing and revising the manuscript. DMF assisted in growing and harvesting the MSC, performed the immunocytochemical staining, and assisted in data collection. JNB provided the MSC and information as to how to culture and harvest them in our laboratory. PAL received funding for the project and provided input during the drafting of the manuscript. All authors read and approved the manuscript.
